# UV-C Light-Based Decontamination of Transvaginal Ultrasound Transducer: An Effective and Fast Way for Patient Safety in Gynecology

**DOI:** 10.3390/jcm14238561

**Published:** 2025-12-02

**Authors:** Tomasz Siewierski, Grzegorz Fibiger, Anna Różańska, Agata Pietrzyk, Estera Jachowicz-Matczak, Dorota Romaniszyn, Jadwiga Wójkowska-Mach

**Affiliations:** 1 Chair of Microbiology, Faculty of Medicine, Jagiellonian University Medical College, ul. Czysta 18, 31-121 Kraków, Poland; tsiewierski@outlook.com (T.S.); a.pietrzyk@uj.edu.pl (A.P.); estera.jachowicz-matczak@uj.edu.pl (E.J.-M.); jadwiga.wojkowska-mach@uj.edu.pl (J.W.-M.); 2 St. Rose Hospital, ul. Skotnicka 230A, 30-394 Kraków, Poland; 3 ARTROMED Orthopedic and Rehabilitation Center, 30-059 Kraków, Poland

**Keywords:** transvaginal ultrasound, UV-C disinfection, infection prevention and control, vaginal microbiota, *Trichomonas vaginalis*

## Abstract

**Background/Objectives**: Transvaginal ultrasound is one of the basic diagnostic methods used during gynecological examination. Lack of care during such an examination can lead to a risk of dangerous infection for both the patient and medical staff. Therefore, a crucial part of the preparation for the test is its proper disinfection. The aim of this study was to test the effectiveness of ultraviolet (UV-C) disinfection of ultrasound probes used in transvaginal examination for the prevention of infections in patients and healthcare personnel in ambulatory care settings. **Methods**: The eradication of microorganisms present (as a microbiota or etiological factors of infections) in the female reproductive tract was tested. They were the following: *Staphylococcus aureus*, *Staphylococcus haemolyticus*, *Streptococcus agalactiae*, *Klebsiella pneumoniae*, *Escherichia coli*, *Acinetobacter baumannii*, *Pseudomonas aeruginosa*, *Candida albicans*, and *Trichomonas vaginalis*. The study consisted of contaminating the ultrasound transducer with microbial strains of known density, then performing disinfection and assessing the number of surviving microbial cells. **Results:** Almost complete eradication of all tested pathogenic microorganisms was achieved, except *T. vaginalis*. But for this protozoa eradication of mobile forms reaches around 80%. **Conclusions:** Although the sample size in our study was limited and statistical significance was not reached, the substantial logarithmic reduction strongly indicates the effectiveness of this method.

## 1. Introduction

The vaginal microbiota is a complex ecosystem of more than 200 microbial species influenced by genes, ethnicity, environmental, and behavioral factors [1]. Its stability and the antagonistic effect of the main component of the vaginal microbiota, i.e., lactic acid bacilli, prevent uncontrolled proliferation of commensal microorganisms and colonization by pathogens, and thus prevent infections [2]. Bacterial vaginosis is observed in 4–75% women, depending on the studied population, although exact prevalence remains difficult to determine [3,4]. In the United States, the prevalence is estimated at 29%, and in Europe it is lower, with a maximum of about 20% in Poland and Norway [4]. Bacterial vaginosis is characterized by a change in the composition of the vaginal flora, with a dramatic reduction in the number of *Lactobacilli* due to a significant overgrowth of anaerobes [5]. Another important etiological factor of infections of the lower genital tract is fungi and the protozoan parasite *Trichomonas vaginalis,* which causes trichomoniasis. Candidal vulvovaginitis results from inflammatory changes in the vaginal and vulvar epithelium, secondary to infection with *Candida* species, most commonly *Candida albicans* [6].

Trichomoniasis is a common global health problem and one of the most prevalent non-viral sexually transmitted diseases worldwide. Infection of the female genital tract can cause various symptoms, including vaginitis, cervicitis, and pelvic inflammatory disease, and infections in men are generally asymptomatic. The relatively mild symptoms and inadequate understanding of epidemiology and public health implications have led to the trichomoniasis being underdiagnosed and underappreciated in the past [7,8]. According to the World Health Organization (WHO) data, there were above 156 million new cases of *T. vaginalis* infections in 2020, exceeding those of other significant sexually transmitted diseases, such as chlamydia, gonorrhea, and syphilis [7,8]. Based on WHO data, the global prevalence of trichomoniasis is estimated at 5.3% and 0.6% in women and men, respectively [9].

The group of special concern, particularly susceptible to infections, is pregnant women. Infections and colonization of the reproductive tract pose a direct threat to the life and health of the fetus and newborn [10]. Symptomatic and asymptomatic bacteriuria are common during pregnancy, with *Escherichia coli* being the most frequent etiological agent in both cases. One of the main causes of complications in both term and low birth-weight infants is infection with group B *Streptococcus agalactiae* [11]. This bacterium is found in the vagina or rectum of approximately 10–30% of all women. GBS (+) pregnant women are more likely to develop symptomatic urogenital tract infections, chorioamnionitis, postpartum endometritis, and sepsis [12]. It has been shown that *T. vaginalis* infection is associated with adverse birth outcomes such as low birth weight, preterm delivery, and premature rupture of membranes. Although rare, the parasite can be transmitted perinatally and cause vaginal or respiratory infections in neonates [7].

Prevention of these infections can therefore be an important factor influencing the well-being of the mother and fetus. The origin of these infections may be both endogenous and exogenous, due to lack of proper infection control in healthcare settings. In addition to direct patient examination, ultrasonography is a basic diagnostic technique in obstetrics and gynecology. Two-dimensional (2D) transvaginal ultrasonography (TVUS) is the most widely used method for assessing the uterine cavity. It is an easy-to-perform, non-invasive, inexpensive, and widely available method for the general population [13]. Given the high risk of these serious infections, rapid, economical, and effective methods of transvaginal probe disinfection are essential. The history of sonography in obstetrics and gynecology dates back to the work of Donald and his Glasgow team, published in the Lancet in 1958. More than fifty years later, it is impossible to imagine practicing obstetrics and gynecology without one of the many forms of ultrasound available today [14].

The safety of the ultrasound procedure itself deserves attention. Although it is by no means an invasive diagnostic method, if some sanitary measures are neglected, it can expose the patient to potential infections and resulting health complications. As in any medical procedure, in the transvaginal ultrasound, the patient’s safety is crucial. Recent reports from around the world show us that ultrasound examination can potentially be a source of infection with dangerous strains of microorganisms. In December 2022, the CDC released a report on hospital-acquired, ultrasound-associated infections. In July 2021, the Virginia Department of Health notified the CDC of eight invasive infections with *Burkholderia stabilis*, a member of the *Burkholderia cepacia* complex (*BCC*), in hospitalized patients who had undergone an ultrasound examination. The CDC, in collaboration with partner healthcare facilities, state and local health departments, and the Food and Drug Administration (FDA), identified 119 *B. stabilis* infections across 10 U.S. states. The source of infection was non-sterile ultrasound gel [15,16]. This issue applies to all types of ultrasound examinations but is particularly concerning in transvaginal ultrasound, as the gel is in direct contact with the patient’s mucous membranes, significantly increasing the risk of infection.

These reports highlight the importance of proper disinfection during this procedure. The hygiene of ultrasound probes remains a major concern in obstetrics and gynecologic ultrasonography. This concern primarily involves the risk of contamination and the need for specific procedures to ensure a high level of protection against infectious diseases, even when disposable probe covers are used [17].

The antimicrobial properties of UV-C have been known since the late 19th century. In the past year, systems and devices for UV-C disinfection using new technology have been intensively studied as an alternative to conventional disinfection methods to eradicate pathogenic microorganisms in hospital settings. Currently, most UV disinfection devices mainly use ultraviolet C (UV-C) radiation with wavelengths between 200 and 270 nm. At certain wavelengths, such as 254 nm, UV-C light can destroy molecular bonds and disrupt DNA or RNA through pyrimidine dimerization, causing the death of a variety of environmental microorganisms [18]. This finding is supported by recent research [16].

The aim of this study was to verify the effectiveness of ultraviolet (UV-C) light disinfection of transvaginal ultrasound probes used in outpatient care to improve the safety of both patients and healthcare workers. The effectiveness of eradicating microorganisms relevant to the etiology of genitourinary tract infections was evaluated.

## 2. Materials and Methods

**Figure 1:**
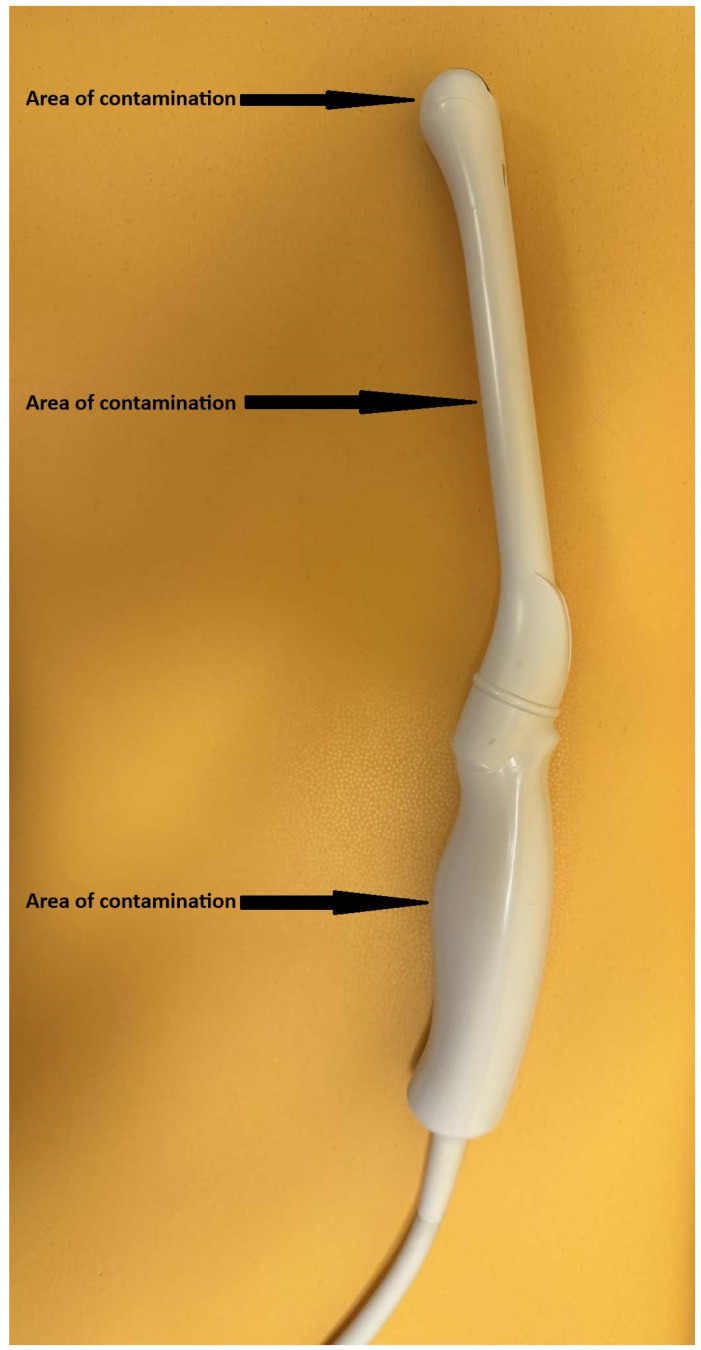
Figure presents E72 endo-vaginal ultrasound transducer that was used in our study.

The study consisted of contaminating the E72 ultrasound transducer with microbial strains of known density, followed by disinfection and evaluation of the number of surviving microbial cells (Figure 1).

### 2.1. Strains and Their Preparation

The strains are as follows:

1. Clinical bacterial strains of *S. aureus*, *S. haemolyticus*, *S. agalactiae*, *K. pneumoniae*, *E. coli*, *A. baumannii*, *and P. aeruginosa* used in the study came from the collection of the Department of Microbiology of the Jagiellonian University Medical College, including strains isolated in 2008–2016 from infections of pregnant or postpartum patients;
2. The reference strain *Candida albicans* ATCC 10231 (American Type Culture Collection, ATCC, Manassas, VA, USA) was used as a control;
3. The reference strain *T. vaginalis* ATCC 30001TM (Manassas, VA, USA) was used a control.

Culture of microorganisms was carried out on appropriate media:

- *S. agalactiae*: Columbia blood agar and TSA (Tryptic-Soy Agar) for other bacteria;
- *Candida albicans*: Sabourauda agar;
- *T. vaginalis*: Diamond liquid medium supplemented with bovine serum and antibiotics (1:1 penicillin-streptomycin mixture).

To prepare bacterial and fungal test suspensions, strains were suspended in growth broth and incubated at 37 °C for 24 h. The suspensions were adjusted to a density of 0.5 MacFarland standard. For precise density determination, each suspension was inoculated onto solid media in six decimal dilutions, incubated for 24 h, and the grown colonies were counted. Parasite cultures were also incubated at 37 °C for 24 h. After 24 h, both test and control samples were evaluated by counting the number of live, motile *T. vaginalis* cells in the Bürker counting chamber.

### 2.2. Probe Contamination and Disinfection Procedure

Using ESwab™ (ESwab combines a COPAN-invented flocked swab with 1 mL of Liquid Amies in a plastic, screw cap tube, COPAN, Brescia, Italy), the tested bacterial and fungal suspensions were spread separately on the two parts of the probe: the handle and the portion that comes into direct contact with the patient during examination. For *T. vaginalis* the entire surface of transvaginal transducers was immersed in the initial suspension of tested strain.

### 2.3. Disinfection Procedure

The Antigermix^®^ S1, a UV-C disinfection chamber designed by Germitec (Ivry-sur-Seine, France), equipped with six low-pressure tubes and two photodiodes to control the UV-C disinfection cycle, was used in this study. The contaminated transducer was placed in the device, and the 90 s disinfection procedure was initiated. All tests were performed in triplicates. The disinfection cycle, programmed according to the manufacturer’s instructions, was 293 mJ/cm^2^ for 90 s at wavelength 254 nm.

### 2.4. Determination of Bacterial and Fungal Suspension Density

Afterwards, samples of bacteria and *C. albicans* from both parts of the probe were collected using a new saline-moistened flocked and seeded onto solid media. The seed plates were placed in the incubator for 24 h, after which the number of colonies that grew was counted. To determine the control suspension density (which may have decreased when transferred from the test surfaces compared with the initial solution), a swab dipped in a 0.5 McFarland’s suspension of each bacterial strain and *C. albicans* was used to make a smear on a 75 cm^2^ plastic plate. After drying, the material was collected using saline-moistened flocked swab and seeded onto solid media in six decimal dilutions. After 24 h of incubation, the colonies were counted and the control density for each bacterial strain was determined. The control sample densities were approximately 1 to 3 log_10_ units lower, likely due to the sampling method involving swap application and the resulting loss of some bacterial cells.

For *T. vaginalis,* the transducer, after treatment, was immersed in fresh supplemented Diamond’s medium and incubated at 37 °C for 24 h. Control samples were not disinfected, and transvaginal transducers were immediately immersed in fresh culture medium after being covered with the parasite suspension. Parasite cultures were also incubated at 37 °C for 24 h. After 24 h, both test and control samples were evaluated by counting the number of live, motile *T. vaginalis* cells in the Bürker counting chamber.

### 2.5. Statistical Analysis

To assess the differences between the transducers and the handles, the non-parametric Wilcoxon signed-rank test for paired samples was applied, comparing the reduction level for each species. All the tests were conducted in RStudio (version 1 December 2024), with R version 4.4.3 (28 February 2025), using a significance level of 0.05.

## 3. Results

A reduction in the density of the culture was obtained in each of the tested microorganisms, excluding *Trichomonas vaginalis*. The densities of Gram-positive cocci decreased by five logarithmic units and for Gram-negative bacilli and Candida albicans decreased by four. In percentage terms, this represents an elimination of between 99.9 and 100.0% of bacterial cells applied to the probe transducer (Table 1). In the case of *E. faecalis,* the reduction in density at the transducer was six logarithmic units (100%), meaning that no bacterial colonies were found after disinfection.

The handle results were slightly better for *S. agalactiae*, *A. baumannii*, and *P. aeruginosa*: reduction level expressed in logarithmic units reached five; however, in percentage terms, it was between 99.96 and 100%. Statistical analysis confirmed that these differences were not significant. The results are presented in Table 1, as the average values for three tests for all bacterial and *C. albicans* strains and additionally, as a consequence of lack of statistical significance for repetitions between the transducer and the handle (transducer vs. handle test 1 *p*-value 0.2228, test 2 *p*-value 0.0656, and test 3 *p*-value 0.6803), as the average for all results, meaning the average for six tests for each species (three transducers and three handle).

For *T. vaginalis*, a 24 h culture in Diamond’s medium was used. The initial cell density was 7.19 × 10^6^ cells/mL and the control density was 8.60 × 10^5^. After 24 h of incubation, the number of viable, motile trophozoites of *T. vaginalis* in the control was on average 1.55 × 10^5^/mL, which means that, even though a reduction expressed in logarithmic units was not observed, there was 82.38% expressed as a percentage decrease in tested suspension.

## 4. Discussion

Our study evaluated the efficacy of ultraviolet disinfection applied to transvaginal ultrasound probes in reducing the risk of pathogenic microorganism transmission and cross-infection. The results showed that UV disinfection achieved 99.88–100% reduction in bacterial suspension for all tested bacteria strains and *C. albicans*. For *T. vaginalis,* the results demonstrated a reduction in trophozoite viability of around 80%. In terms of logarithmic reduction, the magnitude of reduction depended on the bacterial strain, and for *C. albicans*, a reduction of four to six log_10_ was achieved. According to Sauli et al., when evaluating antimicrobial activity, a reduction in bacterial suspension density of ≤2 to <3 log indicates bacteriostatic activity, and reduction of over 3 log indicates bactericidal activity [19]. The method described by Oie et al. can be considered to be proper for testing the antimicrobial efficacy on such surfaces [20]. We tested the effectiveness of UV-C on ultrasound transvaginal transducers as an additional preventive measure in addition to the routinely used transducer cover. Thus, we may treat a reduction of more than 3 log units as an effective way of disinfection. Our study confirms such a level of reduction for all tested microorganisms, with exception of *T. vaginalis*. However, the study of Różańska et al. of the effectiveness of no-touch disinfection method using different clinical, reference, and environmental strains of coagulase-negative staphylococci, *Acinetobacter*, or *C. difficile* shows that substantial differences may be observed even within a given genus [21,22,23]. We performed tests for each microorganism in triplicate, so further studies incorporating a greater number of experimental replicates are required. Increasing the exposure time to UV-C radiation could also bring better results, but 90 s was chosen based on real-world practice. Due to practical context, we tested the efficacy of UV-C disinfection chamber in reducing *T. vaginalis*, which yielded worse results than those observed for tested bacteria and *C. albicans*. However, in our experiment, all surviving bacterial, fungal, or *T. vaginalis* cells were placed in dedicated growth media and incubated. In real life, they would be exposed to the full diversity of the vaginal microbiota. Additionally, the effectiveness of disinfection should be tested for bacteria, mycobacteria, fungi, and viruses. In our study, we compared the density of tested suspensions to control sample density, not initial density. The control sample densities were approximately 1 to 3 log_10_ units lower, likely due to the sampling method involving swap application and the resulting loss of some bacterial cells. This was deliberately performed, but the reported reduction for all tested microorganisms could be even better when compared to the initial suspension density. This is the methodological issue that could be addressed in future studies.

The key point is that the tested disinfection method (UV-C) was applied to a transvaginal ultrasound routinely used by medical personnel. When comparing our findings with benchmark studies conducted on microbial cells in vivo, we observed consistent results [24]. Yang et al. tested a range of microbial groups focusing on antibiotic-resistant bacterial strains (*P. aeruginosa*, *A. baumannii*, Vancomycin-resistant Enterococcus) and ATCC strains of *S.aureus* (MRSA, ATCC 33592) and *M. abscessus* (ATCC 19977). as well as fungal strains (*A. fumigatus*) [25]. Under optimal conditions (5 min of UV exposure at distance of 3 m), Yang et al. achieved a $3\;{log}_{10}$ reduction in the bacterial strains tested. Given the consistency with our results (a reduction of 3–4${\;log}_{10}$ for the tested strains), we can assume that this method is suitable for routine outpatient practice. If not properly disinfected, sonographic transducers can become contaminated not only with bacteria, fungi, or sexually transmitted protozoa like *T. vaginalis*, but also with viruses: HBV (*Hepatitis B virus*), HCV (*Hepatitis C virus*), HIV (*Human immunodeficiency virus*), and HSV (*Herpes simplex virus*), and act as a potential source of infection. For this reason, proper handling, cleaning, and disinfection of transducers are essential [26]. By implementing appropriate safeguards, the safety of both patients and operators can be significantly improved. Studies on endocavitary transducers which undergo high-level disinfection have shown that, despite the use of probe covers, these devices can still transmit pathogens, including HPV. Because manipulation of the probe, gel, and cover can lead to infection and cross-contamination with a variety of pathogens (e.g., MRSA, HBV, HCV, HIV, HPV, and CMV), the probe must be cleaned and disinfected with agents demonstrating bactericidal, fungicidal, and virucidal activity after removal of the protective cover [27].

Disinfection procedures are routinely performed before transvaginal ultrasound examination. It eliminates microorganisms to the level required for this procedure, thereby minimizing the risk of transmission. Since this is not equivalent to destroying all potentially harmful microorganisms, protective probe covers must be used during the examination. However, probe covers alone are not sufficient. For optimal patient protection, effective and rapid disinfection must be combined with the use of protective covers [28].

The advantages of the tested method, in addition to its effectiveness, include ease of use and increase in availability due to the decrease in cost of equipment [25]. It also requires less time and personnel [29]. It can be particularly useful in facilities with a high risk of contamination (high traffic and small space) as it does not require a separate room and qualified staff to perform effective disinfection. Therefore, it represents a convenient and efficient alternative for staff performing examinations in outpatient settings.

Our study had some limitations; first, the number of repetitions was small. As a result, we might perform only a limited analysis of the significance of the results. Another limitation is that the study was restricted to laboratory conditions. As a next step, a clinical study could be conducted aimed at assessing the decontamination procedure with and without UV-C disinfection. However, the value of our study lies in testing the efficacy of UV-C for *T. vaginalis* eradication, which is not routinely evaluated even in current guidelines.

## 5. Conclusions

Our findings suggest that UV-C disinfection could be a safe and effective approach to enhance patient safety during ultrasound procedures in both ambulatory care and hospital settings. However, given the small sample size and the lack of statistical significance, these results should be interpreted with caution. Despite this limitation, the marked logarithmic reduction strongly supports the potential effectiveness of this method. It should also be noted that the elimination of bacteria and fungi was more successful than that of *Trichomonas vaginalis*.

## Figures and Tables

**Table 1:** Microbial viability after UV application. Legend: CFU—colony forming units; n.a.—not available; Log * Log reduction.

Microorganism	Density of Culture [CFU/mL]				Reduction in Density of Culture [%]			Log *
	Initial	After Procedures						
		Control	Transduce	Handle	Transduce	Handle	Transduce and Handle	
*Staphylococcus aureus*	4.20 × 10^7^	1.02 × 10^6^	3.33 × 10^1^	5.00 × 10^1^	100.0	100.0	100.0	5
*Staphylococcus haemolitycus*	2.69 × 10^7^	1.10 × 10^6^	4.00 × 10^1^	3.33 × 10^1^	100.0	100.0	100.0	5
*Enterococcus faecalis*	6.90 × 10^7^	5.50 × 10^6^	6.67 × 10^0^	6.67 × 10^0^	100.0	100.0	100.0	6
*Streptococcus agalactiae*	1.50 × 10^7^	7.70 × 10^5^	1.00 × 10^1^	3.33 × 10^0^	99.9	100.0	100.0	5
*Klebsiella pneumoniae*	8.60 × 10^6^	5.60 × 10^5^	6.67 × 10^0^	3.33 × 10^0^	100.0	100.0	100.0	5
*Acinetobacter baumannii*	1.60 × 10^8^	7.25 × 10^5^	2.67 × 10^1^	0.00 × 10^0^	99.9	100.0	100.0	5
*Pseudomonas aeruginosa*	1.39 × 10^8^	4.40 × 10^5^	7.00 × 10^1^	3.33 × 10^0^	99.9	100.0	99.9	4
*Escherichia coli*	3.75 × 10^7^	2.85 × 10^5^	0.00 × 10^0^	0.00 × 10^0^	100.0	100.0	100.0	4
*Candida albicans*	5.00 × 10^6^	9.50 × 10^4^	0.00 × 10^0^	0.00 × 10^0^	100.0	100.0	100.0	4
*Trichomonas vaginalis*	Density of culture [protozoan cell count/mL]				Reduction in density of culture [%]			Log *
	7.19 × 10^6^	8.60 × 10^5^	1.55 × 10^5^	n.a.	82.4	n.a.	82.4	0

## Data Availability

Data are available from the corresponding author upon reasonable request.
